# Covalent Grafting of Quaternary Ammonium Salt-Containing Polyurethane onto Silicone Substrates to Enhance Bacterial Contact-Killing Ability

**DOI:** 10.3390/polym17010017

**Published:** 2024-12-25

**Authors:** Zihong Pan, Zixu Liu, Sijia Yang, Zhanyu Shen, Yuchen Wu, Yanyu Liu, Jingfan Li, Liang Wang

**Affiliations:** School of Chemistry and Chemical Engineering, Tianjin University of Technology, 391 Binshuixidao, Tianjin 300384, China; pzhyx@stud.tjut.edu.cn (Z.P.); zixu940217663@163.com (Z.L.); yangsijia0709@stud.tjut.edu.cn (S.Y.); szy917505495@stud.tjut.edu.cn (Z.S.); yj20230952wyc@stud.tjut.edu.cn (Y.W.); l1251660224@stud.tjut.edu.cn (Y.L.); ljf1102@stud.tjut.edu.cn (J.L.)

**Keywords:** polyurethane, contact-killing, coating, silicone, implant material

## Abstract

Catheter-associated urinary tract infection (CAUTI) induced by rapid bacterial colonization and biofilm formation on urinary catheters is a key issue that urgently needs to be addressed. To prevent CAUTI, many contact-killing, non-leaching coatings have been developed for the surfaces of silicone catheters. However, due to the chemical inertness of the silicone substrate, most current coatings lack adhesion and are unstable under external forces. Thus, the aim of this study was to develop a surface coating that has both good antibacterial ability and a high affinity toward silicone substrates. To achieve high affinity, a pre-coating layer with abundant surface vinyl groups, named SI-vinyl, was prepared on the silicone substrate by moisture curing using a mixture of α,ω-dihydroxy polydimethylsiloxane and vinyltrimethoxysilane as the painting agent. To endow the surface with contact-killing ability, a series of polyurethanes with different contents of quaternary ammonium salt groups in their main chain and two vinyl end groups were synthesized and covalently grafted onto the surface of SI-vinyl, resulting in corresponding bactericidal coatings with different surface contents of quaternary ammonium salt groups (SI-QAS). Of these bactericidal coatings, SI-QAS-2, with a surface QAS content of 2.1 × 10^16^ N^+^ cm^−2^, was selected as the best coating based on the consideration of stability, compatibility, and antibacterial ability. The SI-QAS-2 coating demonstrated high contact-killing performance, rapidly inactivating 72.8%, 99.9%, and 98.9% of Escherichia coli, Staphylococcus aureus, and Pseudomonas aeruginosa within 30 min. Furthermore, even after being exposed to a high concentration of bacteria (10^6^ CFU/mL) for 4 days, the SI-QAS-2 coating still maintained a high bactericidal ratio of over 80%. In summary, we developed a novel contact-killing coating that reduces the risk of bacterial infections caused by catheter implantation, demonstrating that it has high affinity toward silicone substrates, excellent contact-killing efficiency, a facile preparation method, and potential for further application.

## 1. Introduction

Because of its flexibility, chemical inertness, and biocompatibility, silicone rubber is widely used as the main component of many implantable medical devices, including catheters, scaffolds, and plastic surgery materials [1,2,3]. However, a critical yet unresolved problem in this field is the almost inevitable bacterial infection induced by the embedded device. Microorganisms can attach to and grow on the surfaces of these indwelling silicone devices, eventually forming biofilms [4,5]. These biofilms are often resistant to antibiotic treatment due to reduced antimicrobial uptake, which results from the chemical and architectural structure of the exopolysaccharide film and causes changes in the metabolic state of the bacteria [6,7]. Such surface colonization is particularly problematic for patients with central venous catheters, as biofilm detachment delivers a bolus of microorganisms intravenously to the patient, diminishing the ability of the immune system to clear the infection [8,9]. Furthermore, there is a high risk that bacteria infecting peri-implant tissues will develop antibiotic resistance via point mutation when using antibiotic monotherapy [10,11]. To reduce the risk of infections, some physicians have resorted to antibiotic prophylaxis in clinical treatment. However, concerns over cost, side effects, and the emergence of antibiotic-resistant pathogens exist [12]. Overall, these issues emphasize the need for alternative methods to prevent the bacterial colonization of and biofilm formation on implantable devices.

To prevent bacterial attachment, antifouling coatings inhibit biofilm formation by increasing resistance to the initial adhesion of bacteria to surfaces. The recent introduction of hydrophilic zwitterionic polymers onto medical device surfaces has proven to be an effective solution for resisting fouling [13,14]. Zwitterionic polymers, including poly(carboxybetaine methacrylate), poly(2-methacryloyloxyethyl phosphorylcholine), and poly(sulfobetaine methacrylate), are able to strongly bind a large number of water molecules via electrostatically induced hydration, forming a stable hydration layer on the material surface that effectively prevents the initial attachment of bacteria and subsequent biofilm formation [15,16,17]. Another approach is to use coatings with low surface energy to improve surface antifouling and antibacterial adhesion ability. Fluorinated polymers, such as PTFE particles [18], perfluoropolyether [19], and polyvinylidene [20], are commonly involved in these strategies and can impart a hydrophobic and bio-inert surface. In addition, the hydrophobicity of these polymeric coatings can be enhanced to achieve superhydrophobicity through their micro/nanosurface patterns. This further enhances the antifouling ability of these coatings [21,22,23]. However, due to their lack of bactericidal activities, these passive-defense antifouling surfaces are still contaminated by bacteria after prolonged exposure in complex internal environments, ultimately leading to catheter-associated infections [24,25,26]. To compensate for the shortcomings of antifouling surfaces, many coatings with sterilization functions have been developed for use on implant materials [27,28,29]. In terms of their antibacterial activity, these coatings can be divided into two categories: release-killing and contact-killing. As an example of release-killing strategies, antibacterial coatings containing silver or antibiotics have been applied to urinary catheters and were the first to be commercialized [30,31,32]. However, the release-killing strategy has its own shortcomings. Its effectiveness is reduced as the loaded biocide is released [33]. Furthermore, side effects caused by the released biocide are usually unavoidable [34]. For example, the continuous release of antibiotics can lead to the proliferation of drug-resistant bacteria, while the in vivo accumulation of Ag^+^ results in physiological toxicity. In contrast to the release-killing strategy, contact-killing surfaces are ideally non-leaching and can retain their antibacterial ability for relatively long durations. They also inhibit pathways for the growth of drug-resistant bacteria. Owing to their broad-spectrum antibacterial activity, good chemical stability, and low cost and the design flexibility of their molecules, quaternary ammonium salt compounds have been widely studied as bactericidal agents [35,36]. In particular, small-molecule quaternary ammonium salts with five to seven carbon atoms have been reported to have high antibacterial activity [37]. In the field of antibacterial surfaces, quaternary ammonium salt-derived compounds are also preferred for the preparation of contact-killing coatings. For example, Becker et al. reported a series based on polyurethane with quaternary ammonium salt groups in the main chain [38,39]. The catheter produced from this QAS-containing polyurethane had a fast contact-killing ability and achieved the long-term inhibition of bacterial film formation. In another study, contact-killing and antifouling functionalities were integrated into the surface coating of the silicone catheter [40]. A hydrophilic and uniform polymeric coating was developed for the commercial catheter surface via mussel-inspired chemistry, using amino-rich quaternary ammonium polyethyleneimine, dopamine, and poly(carboxylbetaine-co-dopamine methacrylamide) as the bactericidal linker, adhesive, and antifouling components, respectively. This coating could effectively resist bacterial attachment and inactivate attached bacteria with over a 99.9% kill efficiency. Although significant advancements have been made in the field of contact-killing coatings, further improvement is still needed regarding the adhesion ability, stability, and antibacterial performance of these coatings on implant materials, especially inert silicone substrates.

In this work, a series of novel contact-killing coatings were developed for silicone substrates via two processes. Firstly, the silicone substrates were pretreated with α,ω-dihydroxy polydimethylsiloxane (PDMS) and vinyltrimethoxysilane (VTMS) using the simple dip-coating and moisture cure methods to produce a vinyl-rich silicone layer. Secondly, a class of polyurethanes with different QAS contents and two vinyl end groups (QAPU-vinyl) were prepared and grafted onto the vinyl-rich silicone layer under UV irradiation. Thus, a series of SI-QAS coatings with varying QAS contents were obtained. In this strategy, the pre-coated vinyl-rich silicone layer (SI-vinyl) layer acted as a functional interfacial agent between the silicone substrate and QAPU-vinyl. It had a structure, a composition, and mechanical properties that were highly similar to those of silicone substrates, allowing it to firmly adhere to their surface. On the other hand, its abundant surface vinyl groups could serve as post-modification sites, promoting the grafting of QAPU-vinyl. In addition, when covalently grafted onto the surface of SI-vinyl, the synthesized QAPU-vinyl endowed the silicone substrate with contact-killing ability. Finally, the bactericidal ability of SI-QAS was fully assessed in short-term contact-killing and long-term sterilization experiments, using Escherichia coli (*E. coli*), Staphylococcus aureus (*S. aureus*), and Pseudomonas aeruginosa (*P. aeruginosa*) as bacterial models. The surface chemical composition and morphology of these SI-QAS coatings, as well as the surface QAS content, were characterized using X-ray photoelectron spectroscopy (XPS), a scanning electron microscope (SEM), and fluorescein sodium staining.

## 2. Materials and Methods

### 2.1. Materials

2-Butyl-2-ethyl-1,3-propanediol (BEPD) was obtained from TCI (Shanghai, China). Diglycolamine was purchased from Merck Ltd. (Beijing, China). Diphenyl(2,4,6-trimethylbenzoyl)phosphine oxide (TPO) was obtained from Heowns (Tianjin, China). Stannous octoate (Sn(Oct)_2_), 4-vinylbenzyl chloride, hexamethylene diisocyanate (HDI), N-methyldiethanolamine (MDEA), dibutyltin dilaurate (DBTD), CDCl_3_, and VTMS were all purchased from Energy Chemical (Shanghai, China). PDMS was purchased from Zhejiang Rongli High-tech Materials Co., LTD (Tongxiang, China). K_2_CO_3_, NaCl, and other reagents were purchased from the Tianjin Damao Chemical reagent factory (Tianjin, China). All the chemicals were used without further purification.

### 2.2. Synthesis of End-Capping Agent N-Bis(vinylbenzyl) Diglycolamine

4-Vinylbenzyl chloride (9.2 g, 60 mmol) was added into 50 mL of ethanol solution containing diglycolamine (3.2 g, 30 mmol) and K_2_CO_3_ (8.3 g, 60 mmol). The mixture was stirred at 50 °C under the protection of nitrogen for 24 h. Afterwards, the reaction mixture was filtered to remove KCl. The obtained filtrate was concentrated, and the crude product was obtained as a yellow oil. The crude product was further purified using silica gel column chromatography, with an eluent composed of an 8/2 volume ratio of ethyl acetate and petroleum ether. Finally, 7.6 g of pure *N*-bis(vinylbenzyl) diglycolamine was collected with a yield of around 75%.

### 2.3. Synthesis of QAPU-Vinyl

Using BEPD and MDEA as monomers, HDI as a condensing agent, and *N*-bis(vinylbenzyl) diglycolamine as a capping agent, PU-vinyl polymers were prepared via polycondensation. By tuning the feeding molar BEPD–MDEA ratio from 9:1 to 8:2, 7:3, and 6:4, four PU-vinyl polymers were obtained, which were named PU-vinyl-1, PU-vinyl-2, PU-vinyl-3, and PU-vinyl-4, respectively. Taking PU-vinyl-2 as an example, the synthesis is briefly described below. BEPD (3.8 g, 24 mmol) and MDEA (0.7 g, 6 mmol) were dissolved in 40 mL of toluene, and the majority of solvent was distilled out at 110 °C to remove trace water from the monomers. After cooling to 80 °C, 50 mg of Sn(Oct)_2_ was added to the solution as a catalyst, and HDI (5.6 g, 33 mmol) was then slowly added to the solution. Under nitrogen protection, this reaction system was stirred at 80 °C for 3 h. The end-capping agent, *N*-bis(vinylbenzyl) diglycolamine, (1.3 g, 4 mmol) was then added into the reactant mixture followed by continuous stirring for another 1 h. PU-vinyl-2 was collected and purified using a dissolving–precipitation method, with tetrahydrofuran as a good solvent and petroleum ether as a poor solvent, and then dried for 24 h at 40 °C under vacuum. To introduce QAS groups into the polyurethane backbone, the PU-vinyl-1, -2, -3, and -4 polymers were further treated with dimethyl sulfate to produce QAPU-vinyl-1, -2, -3, and -4. Briefly, PU-vinyl-2 (2.3 g) and excess dimethyl sulfate (0.59 g, 5 mmol) were dissolved in 10 mL of chloroform and refluxed at 50 °C for 24 h. The reaction mixture was washed with 10 mL of water to remove excess dimethyl sulfate and poured into a sufficiently large volume of petroleum ether. The crude product was purified by dialysis and then freeze-dried to obtain light-yellow powder-like QAPU-vinyl-2.

### 2.4. Preparation of SI-Vinyl

PDMS (3.0 g), VTMS (2.0 g), and DBTD (10.0 mg) were dissolved in 10 mL of chloroform to prepare the painting solution. The silicone film samples (1.0 cm × 1.0 cm with 0.1 cm thickness) were then immersed in the painting solution for approximately 10 s, slowly withdrawn, and placed on a Teflon dish. After allowing the solvent to evaporate naturally, SI-vinyl was formed through moisture curing at room temperature for 24 h.

### 2.5. Surface Grafting of QAPU-Vinyl

QAPU-vinyl-1, QAPU-vinyl-2, and QAPU-vinyl-3 were used as raw materials to prepare three QAS-containing coatings, namely SI-QAS-1, SI-QAS-2, and SI-QAS-3, using a UV-curing method. Taking SI-QAS-2 as an example, the steps involved in the preparation method for this coating are described in the following. QAPU-vinyl-2 (1.0 g) was dissolved in chloroform (10 mL), and TPO (100 mg) was then added into this solution. SI-vinyl was immersed in the above solution for 10 s and slowly withdrawn, leaving a thin liquid film on its surface. After the solvent had mostly evaporated, surface grafting of QAPU-vinyl-2 was carried out in a UV-curing box for 3 min to produce SI-QAS-2. To remove noncovalently attached QAPU-vinyl-2, the SI-QAS-2 silicone film sample was washed with chloroform three times. After vacuum drying at 45 °C for 24 h, clean SI-QAS-2 was obtained and prepared for subsequent experiments. Other SI-QAS samples were also prepared in the same manner.

### 2.6. Determination of Surface QAS Content on SI-QAS

SI-QAS samples (1.0 cm × 1.0 cm) were immersed in a solution of fluorescein sodium (1.0 wt% in distilled water) for 10 min. Unreacted fluorescein molecules were removed by washing with distilled water, and the samples were then placed in 3 mL of sodium chloride saturated solution under ultrasound for 15 min to undergo exchange with bound fluorescein molecules. Then, 0.45 mL of saturated NaHCO_3_ solution was added into the extraction solution. The UV absorbance of the obtained solution was recorded at 501 nm. The concentration of the released fluorescein sodium was calculated according to the standard curve of fluorescein sodium. Assuming that the number of the released fluorescein sodium molecules was equal to the number of QAS groups on the surface of SI-QAS, the QAS content could be calculated from the concentration of fluorescein sodium and expressed as the number of QAS groups per square centimeter (N^+^/cm^2^). Data were collected as the average number of five parallel experiments for each sample and are presented as the mean ± standard deviation (SD).

### 2.7. Contact-Killing Abilities of SI-QAS

The contact-killing abilities of SI-QAS were determined according to the ISO 22196 standard [41] by quantifying the loss of viability of bacteria held in close contact with the film sample for 30 min at 37 °C. *E. coli*, *P. aeruginosa*, and *S. aureus* were selected as the bacterial models of Gram-negative, Gram-positive, and infectious bacteria. The silicone film without the antibacterial coating was used as a control group. The experimental details were as follows. The film samples (1.0 cm × 1.0 cm), sterilized by ultraviolet (UV) irradiation, were placed into sterile Petri dishes. Bacterial suspensions (20 μL) containing 10^6^ CFU/mL bacterial cells were pipetted onto the film samples and then covered with a piece of square polyethylene film (0.5 cm × 0.5 cm). These samples were incubated at 37 °C under a relative humidity of above 90% for 30 min. After incubation, each film sample was placed into 5 mL of phosphate buffer solution (pH = 7.4) and then sonicated to release the adhered bacterial cells into the solution. Then, 50 µL of the above bacterial dispersion was inoculated onto LB agar plates. After incubation at 37 °C for 16 h, colonies that had grown on the plates were counted, and the loss of viability was calculated and presented as the percentage of dead bacteria compared to the control group. Results were reported as mean ± SD (*n* = 5) for each sample.

### 2.8. Bacterial Morphologies on SI-QAS-2

The bacterial morphologies on the surface of SI-QAS were observed using an SEM. The film samples for the SEM were prepared as follows. Bacterial suspension (20 μL) containing 10^6^ CFU/mL bacterial cells was dripped onto the SI-QAS films, covered by a polyethylene film (0.5 cm × 0.5 cm), and incubated at 37 °C for 30 min. After gently washing with phosphate buffer solution three times, the bacterial cells on the SI-QAS were fixed using 2.5% glutaraldehyde aqueous solution for 12 h. The obtained film samples were gradually dehydrated with a series of ethanol/water mixed solutions, with the ethanol concentrations ranging from 10, 30, 50, 70, and 90 vol% to pure ethanol, and then freeze–dried under vacuum. Finally, the as-prepared film samples were observed using the SEM under an electron acceleration voltage of 10.00 kV.

### 2.9. Long-Term Bactericidal Activity of SI-QAS-2

In this experiment, the silicone samples without antibacterial coating were used as the control group, while *E. coli*, *S. aureus* and *P. aeruginosa* were still employed as bacterial models. Prior to the experiment, the control and SI-QAS-2 film samples (1.0 cm × 1.0 cm) were sterilized under UV and separately placed into a well in a 24-well plate. Bacterial suspension (0.50 mL) containing 10^6^ CFU/mL bacterial cells was then added into each well. These samples were incubated for five days at 37 °C and a relative humidity above 90%. During this period, the bacterial suspension in each well was replaced daily by a freshly prepared bacterial suspension of the same volume and bacterial concentration and, at the same time, the number of surviving bacteria was determined using the colony counting method. Data were collected as the average number of four parallel experiments for each sample and are presented as the mean ± SD.

### 2.10. Characterization

Fourier-transform infrared spectroscopy (FT-IR) was performed using a Bio-Rad 6000 (Thermo Electron, Waltham, MA, USA) spectrometer, and the samples were characterized by pressing KBr pellets. ^1^H NMR and ^13^C NMR spectra were obtained using a Bruker AV-400 spectrometer (400 MHz; Bruker, Freemont, CA, USA) with CDCl_3_ as solvent. The molecular weight of the synthesized DC was determined using LC-20AP (SHIMADZU, Kyoto, Japan). Water contact angle (WCA) measurements were performed using a contact angle goniometer (SZ-CAMA1, Shanghai Sunzern Instrument Co. Ltd., Shanghai, China)). The surface chemical information of the film samples was characterized using XPS (ESCA-LAB250Xi, Thermo Scientific, Waltham, MA, USA). The topography of the silicone surfaces before and after modification was observed using an SEM instrument (Carl Zeiss AG, MIRA3-GM, Oberkochen, Germany).

## 3. Results

### 3.1. Synthesis of QAPU-Vinyl

#### 3.1.1. Synthesis of End-Capping Agent *N*-Bis(vinylbenzyl) Diglycolamine

**Scheme 1:**
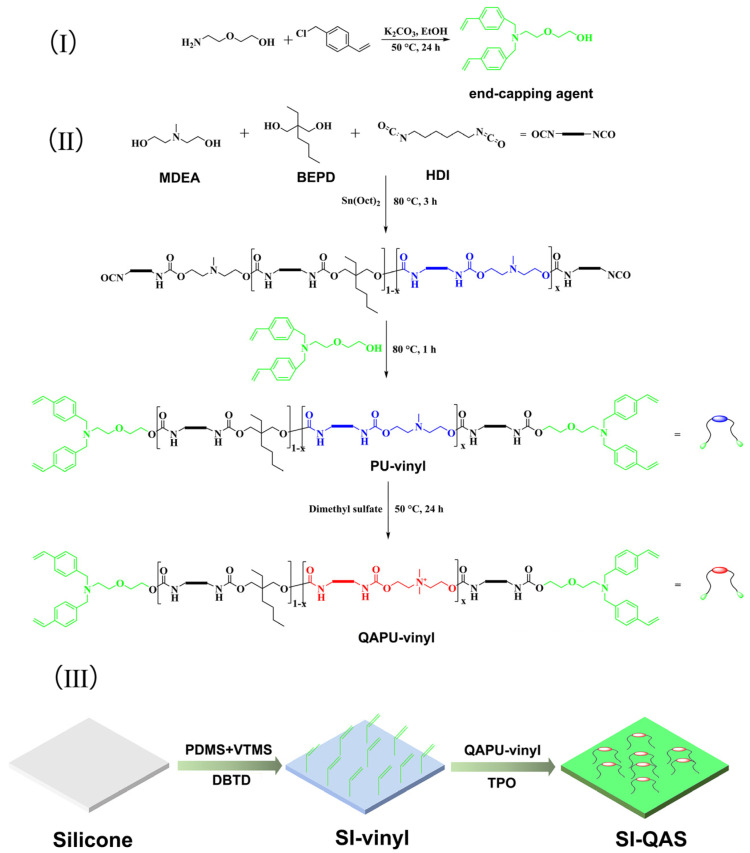
Schematic illustrations of the synthesis of *N*-bis(vinylbenzyl) diglycolamine (I) and QAPU-vinyl (II) and the preparation of SI-QAS (III).

To obtain polyurethane with a modifiable end group, *N*-bis(vinylbenzyl) diglycolamine, which contains two vinyl groups and one hydroxyl group, was synthesized and used as a functional end-capping agent. The synthetic route for DC is shown in Scheme 1(I). As shown in Scheme 1(I), nucleophilic substitution reactions took place between diglycolamine and 4-vinylbenzyl chloride, producing *N*-bis(vinylbenzyl) diglycolamine with a yield of 75%.

The chemical structure of *N*-bis(vinylbenzyl) diglycolamine was confirmed using ^1^H NMR, ^13^C NMR, and MS spectrometry, and the spectra are shown in Figures S1–S3. The signals at 5.24 and 6.77 ppm (g, h) in Figure S1 are assigned to protons from the vinyl groups, while the signals at 3.72, 3.62, 3.53, 2.72, and 3.66 ppm (a, b, c, d, and i) are contributed by the protons from methylene groups at different chemical positions. The peaks (e and f) generated by protons in the benzene ring are located at 7.39 ppm. Combined with the results of ^13^C NMR and MS spectroscopy (Figures S2 and S3), it is confirmed that the end-capping agent *N*-bis(vinylbenzyl) diglycolamine with the predetermined structure was successfully synthesized.

#### 3.1.2. Characterization of QAPU-Vinyl

Prior to quaternization, a series of low-molecular-weight PU-vinyl polymers were prepared using MDEA and BEPD as the diol monomers and *N*-bis(vinylbenzyl) diglycolamine as the end-capping agent using a feeding ratio with excess HDI (HDI/diol monomers = 1.1:1) (Scheme 1(II)). In the PU-vinyl polymer, the tertiary amine groups from MDEA acted as quaternization sites, while pendant long-chain alkane from BEPD acted as a soft segment that could also enhance adhesion to the silicone substrate surface. Following polycondensation, excess isocyanate groups were capped using *N*-bis(vinylbenzyl) diglycolamine to obtain vinyl end groups. To remove the excess end-capping agent, the obtained PU-vinyl was dissolved in THF and precipitated thrice using a sufficiently large amount of petroleum ether. By adjusting the feed ratio of MDEA content in the diol monomers from 10 to 20, 30, and 40 mol%, four PU-vinyl samples with different numbers of tertiary amine groups were obtained. The molecular weights of these PU-vinyl polymers were determined using GPC and are listed in Table 1. As shown in Table 1, upon increasing the MDEA feeding content from 10 mol% to 40 mol%, the Mw of the PU-vinyl was slightly reduced from 11,070 to 8380. MDEA is a tertiary amine that may contain trace water and secondary amines. These impurities may each react separately with HDI, leading to unnecessary HDI consumption and ultimately resulting in a decrease in molecular weight.

**Figure 1:**
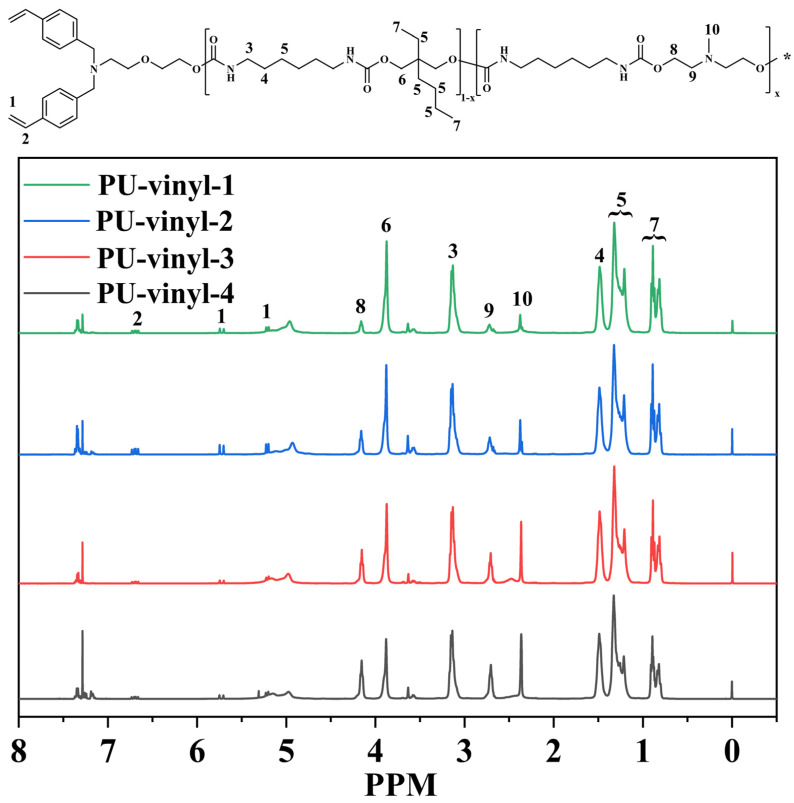
^1^H NMR spectra of PU-vinyl-1, -2, -3, and -4.

The ^1^H NMR spectra of the PU-vinyl series are shown in Figure 1. Referring to the ^1^H NMR spectrum of *N*-bis(vinylbenzyl) diglycolamine, it can be concluded that the multi-peaks at 6.70 ppm (2), 5.73 ppm (1), and 5.20 ppm (1) are hydrogen signals contributed by the CH_2_ = CH- end groups. The signals at 3.13 ppm (3) and 1.48 ppm (4) are attributed to the -CH_2_- groups at the α and β positions beside the urethane groups in the HDI units. The single peak at 3.88 ppm (6) is contributed by the -CH_2_- groups in the BEPD unit connected to the oxygen side of the urethane groups. Similarly, the broad peak at 4.16 ppm (8) is due to the -CH_2_- groups in the MDEA unit. The proton signal at 2.72 ppm (9) is attributed to the -CH_2_- groups connected to the N atom in tertiary amine, while the signals of the methylene groups (5) that are not connected to heteroatoms in HDI and BEPD units overlap and form a multi-broad peak in the range of 1.13 ppm to 1.41 ppm. The methyl hydrogen atoms originating from the MDEA and BEPD units generated signals at 2.37 ppm (10) and 0.85 ppm (7), respectively. From Figure 1, it can be seen that as the MDEA feeding ratio was increased, the signals (8, 9, and 10) related to the MDEA unit also increased. The percentage of MDEA unit to diol units for each sample can be quantitatively calculated from the peak areas of peaks 6 and 8, and the calculated MDEA percentages are basically consistent with the feeding ratio (Table 1). In short, the ^1^H NMR characterization reveals information mainly about the group types, unit ratio, and end-group structure of the PU-vinyl samples, confirming the successful synthesis of PU-vinyl.

Following methylation with dimethyl sulfate, the tertiary amine groups in the MDEA units were converted into quaternary amine groups to generate the target QAPU-vinyl series comprising QAPU-vinyl-1, QAPU-vinyl-2, QAPU-vinyl-3, and QAPU-vinyl-4 (Scheme 1(II)). Excess dimethyl sulfate and the by-product of sulfuric acid were cleared via dialysis, and the purified QAPU-vinyl was finally collected by freeze-drying. The obtained QAPU-vinyl polymers were more hydrophilic and easily adsorbed moisture from the air, becoming viscous. FT-IR was used to monitor the changes in functional groups during the reaction process from the monomers MDEA, HDI, and BEPD to QAPU-vinyl, and the results are shown in Figure S5. The absorptions at 2931 and 2862 cm^−1^ appear in all spectra and are contributed by C-H stretching vibration in the methyl and methylene groups. A peak at 2267 cm^−1^ that only appears in the FT-IR spectrum of HDI is attributed to the NCO groups. Following polycondensation, the NCO peak at 2267 cm^−1^ disappears, while a strong absorption peak emerges at 1698 cm^−1^ in the PU-vinyl-2 spectrum. This new peak is characteristic for the absorption of urethane groups, indicating the successful synthesis of PU-vinyl. Comparing the FT-IR spectra of PU-vinyl and QAPU-vinyl, a new absorption peak observed at 1012 cm^−1^ is attributed to the stretching vibration of C-N in the quaternary amine group, indicating the formation of quaternary amine groups. Notably, a broad absorption appears at around 3339 cm^−1^ in the QAPU-vinyl spectrum, which is due to the absorption of water, consistent with its strong hygroscopicity.

### 3.2. Preparation and Optimum Seeking of SI-QAS Coatings

Prior to surface QAS grafting, the silicone substrate was pretreated with a mixed coating solution containing PDMS and VTMS (weight ratio of 3:2). As they had a similar composition and properties to the silicone substrate, the newly formed SI-vinyl layer exhibited extremely high stability and compatibility. Then, under UV irradiation, the four QAPU-vinyl samples were grafted onto SI-vinyl to form four SI-QAS coatings (Scheme 1(III)). Of these, the SI-QAS-4 coating did not adhere well to the silicon substrate and exhibited severe peeling. QAPU-vinyl-4, which contained the highest content of QAS in its main chain, had the strongest crystallinity and highest hydrophilicity. These properties were deeply incompatible with the hydrophobic SI-vinyl. Also, it had poor solubility in the coating solvent. These factors greatly reduced its affinity with the silicone surface, resulting in extremely low grafting efficiency and poor adhesion to SI-vinyl. Therefore, the SI-QAS-4 coating was excluded from further consideration in this study.

**Figure 2:**
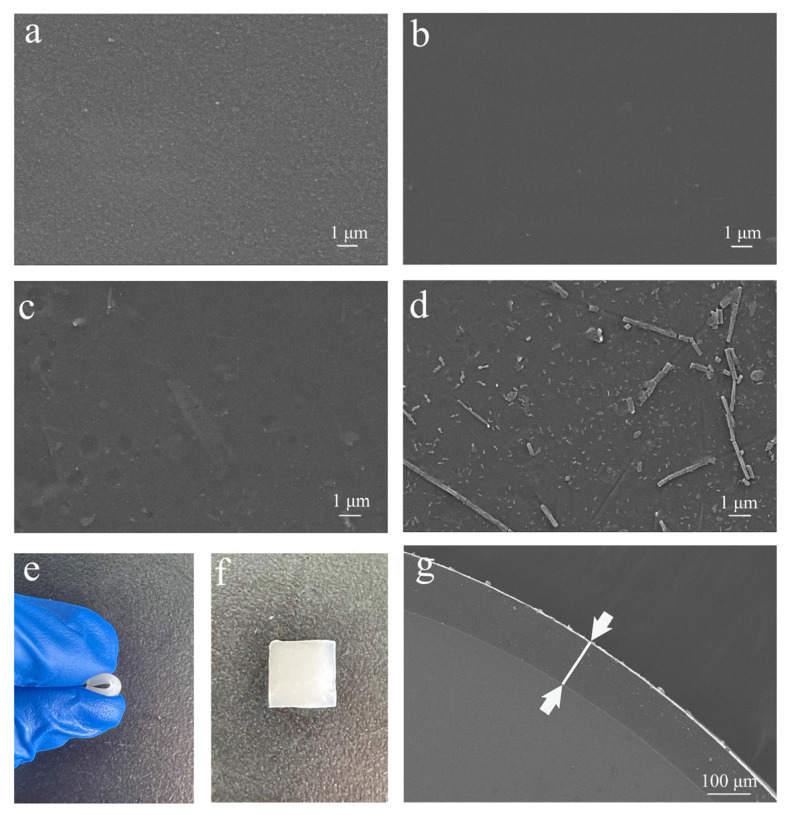
SEM images of (**a**) SI-vinyl, (**b**) SI-QAS-1, (**c**) SI-QAS-2, and (**d**) SI-QAS-3. (**e**,**f**) Comparison image of SI-QAS-2 before and after bending. (**g**) SEM image of sectional view of SI-QAS-2.

The surface morphologies of the remaining three SI-QAS-1, -2, and -3 samples were characterized using an SEM and are shown in Figure 2. In Figure 2a, the SI-vinyl pre-coating presents a micrometer-level rough surface morphology. These solid nuclei might have been formed during the moisture curing process from the cross-linking reaction between PDMS and VTMS. Figure 2b shows the surface morphology of SI-QAS-1. Compared to the SI-vinyl, SI-QAS-1 is obviously smoother. Normally, low-molecular-weight polymers have good fluidity and can act as leveling agents in surface coating systems and may explain this smoothing phenomenon. Notably, no wrinkles or visible signs of detachment were observed on the SI-QAS-1 coating surface, suggesting good compatibility between the grafting layer and SI-vinyl. SI-QAS-2 has inferior surface uniformity to SI-QAS-1 (Figure 2c), exhibiting some spots indicating aggregation. This is due to the increased QAS content in SI-QAS-2, which led to the formation of QAS crystals during QAPU-vinyl grafting. As for SI-QAS-3, there is even more severe crystallization of QAS (Figure 2d). QAS crystals aggregated on the surface of SI-QAS-3, forming numerous strip-shaped crystalline regions, indicating that QAPU-vinyl-3 has poor film-forming abilities and may not be suitable as for use as a coating. Because of this, SI-QAS-3 was also excluded from further study. Coating stability is a critical factor that directly affects the performance and safety of catheters. The stability of SI-QAS-2 on a silicone substrate depends on its adhesion ability and flexibility in adapting to the substrate [42]. To evaluate the coating stability, a sample of SI-QAS-2 was cut into small square pieces for extreme bending under natural conditions. As shown in Figure 2e,f, even when folded over 90°, the SI-QAS-2 coating still adhered well to the substrate, without any wrinkling or peeling. Figure 2g is a sectional view of the SI-QAS-2 coating. From this figure, it can be seen that the SI-QAS-2 coating, composed of the pre-coated SI-vinyl layer and the grafted QAS layer, adhered well to and was uniformly coated onto the silicone substrate, and the total thickness was about 100 µm.

XPS was used to reveal the surface chemical composition of SI-vinyl and SI-QAS-2. The wide-scan spectrum of SI-vinyl (Figure S6a) routinely shows Si 2p, Si 1s, C 1s, and O 1s peaks at 103, 154, 285, and 533 eV, respectively. In contrast, the appearance of the N 1s peak at 401 eV in the wide-scan spectrum of SI-QAS-2 (Figure S6a) is contributed by the surface grafting of QAPU-vinyl. The C 1s spectrum of SI-vinyl (Figure S6b) is curve-fitted to two peaks located at 284.6 and 285.0 eV, respectively. The peak at 285.0 eV is contributed by the unsaturated carbon (C=C) from the surface vinyl groups, indicating that vinyl groups could be effectively introduced onto the surface of the silicone substrate by pre-coating with PDMS and VTMS. The C 1s spectrum of SI-QAS-2 is fitted to four peaks (Figure S6c). The peaks at 284.6, 285.2, and 286.5 eV are, respectively, attributed to C-C, C-N, and C-O originating from the SI-QAS-2 coating, while the peak at 289.6 eV represents the characteristic binding energy from the urethane carbon. It is worth noting that the appearance of a peak located at 402.8 eV in the N 1s spectrum of SI-QAS-2 (Figure S6d) originates from the binding energy of N atoms in the QAS group, providing direct evidence for the presence of QAS groups on the surface of SI-QAS-2. In a word, the above results demonstrate the successful surface grafting of QAPU-vinyl.

**Figure 3:**
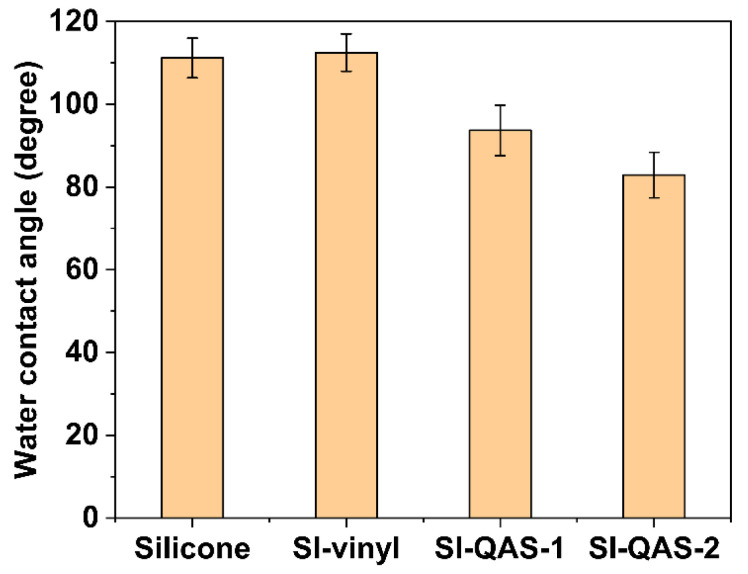
WCAs of silicone substrate, SI-vinyl, SI-QAS-1, and SI-QAS-2.

WCA is an important parameter for measuring the wetting ability of water on solid surfaces. Here, it was used to monitor the changes in the hydrophilicity of the silicone surface before and after QAPU-vinyl grafting. Because silicone and SI-vinyl had similar structures and components, being mainly composed of dimethylsiloxane units, their WCAs were also very similar, at 111.2° and 112.5° (Figure 3), respectively. QAS groups typically have good water solubility and can bind to water molecules through electrostatic attraction. Hence, it was expected that the resulting surfaces would be highly hydrophilic after surface modification with QAPU-vinyl. However, the WCAs of SI-QAS-1 and SI-QAS-2 decreased to 93.7° and 82.9°, respectively (Figure 3). Unlike small-molecule QAS, polymers have a random coil structure, and the QAS groups in QAPU-vinyl could be encapsulated by the hydrophobic BEPD and HDI segments. Therefore, these two SI-QAS coatings did not exhibit the expected high hydrophilicity. Regardless, the decrease in the WCA indicated an increasing content of surface hydrophilic groups, which implied the existence of surface QAS groups.

Fluorescein sodium staining was used to quantitatively determine the content of QAS groups on the SI-QAS surface. Firstly, the anions in the QAS groups on the SI-QAS coating surfaces were replaced by fluorescein molecules in the fluorescein sodium solution. Secondly, by immersing the coatings in saturated saline water, the fixed fluorescein molecules were released back into the aqueous phase. The amount of the released fluorescein molecules in the aqueous phase was measured using UV spectroscopy. The corresponding UV and calibration curves are provided in the Supporting Information (Figure S4). The surface QAS contents of SI-QAS-1 and SI-QAS-2 were finally calculated according to the above UV results as 1.2 × 10^16^ and 2.1 × 10^16^ N^+^/cm^2^, respectively. No QAS groups were detected on the surface of the control sample (SI-vinyl). QAS content is the key factor determining germicidal efficiency [43]. Many studies have revealed that the lower limit of the QAS surface content for killing *E. coli* and *S. aureus* is 10^14^ N^+^/cm^2^. Consistent with this, the surface QAS contents of SI-QAS-1 and SI-QAS-2 were both higher than this value.

### 3.3. The Assessment of the Bactericidal Abilities of the SI-QAS Coatings

**Figure 4:**
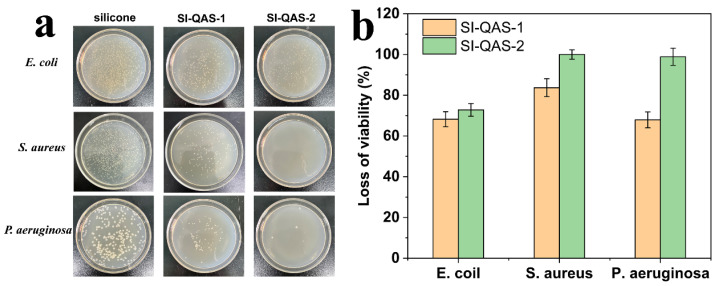
Images of bacterial colonies on LB agar plates for *E. coli*, *S. aureus*, and *P. aeruginosa* after incubating with silicone, SI-QAS-1, and SI-QAS-2 for 30 min (**a**). Loss of viability of *E. coli*, *S. aureus*, and *P. aeruginosa* after incubating with SI-QAS-1 and SI-QAS-2 for 30 min (**b**).

Due to their excellent film-forming ability and sufficient surface QAS content, SI-QAS-1 and SI-QAS-2 were selected for further research on their contact-killing performance. The silicone substrate was used as the control group, and three main bacteria-contaminated pathogens, *E. coli*, *P. aeruginosa*, and *S. aureus*, were used as bacterial models. These bacteria, at 10^6^ CFU/mL, were allowed to come into contact with the SI-vinyl, SI-QAS-1, and SI-QAS-2 coatings for 30 min. Figure 4 shows the photos of LB agar plates for the three samples. Compared to SI-vinyl (left column), SI-QAS-1 (middle column) and SI-QAS-2 (right column) presented a significant decrease in the colony number of all three bacteria. Especially in the case of SI-QAS-2, it was difficult to find a viable bacterial colony in the LB agar plates of *P. aeruginosa* and *S. aureus*. The quantitative loss of the viabilities of these three bacteria are shown in Figure 4b. After 30 min, the loss of the viability of *E. coli*, *P. aeruginosa*, and *S. aureus* following contact was 68.2%, 83.7%, and 67.9%, respectively, for SI-QAS-1 and 72.8%, 99.9%, and 98.9%, respectively, for SI-QAS-2. Due to its high surface QAS content, SI-QAS-2 exhibited much higher bactericidal ability than SI-QAS-1. In addition, these two SI-QAS samples, especially the SI-QAS-1 sample with a lower QAS content, showed higher bactericidal ability against *S. aureus* than other bacteria. Owing to their thicker membrane and lower negative charge content, *E. coli* and *P. aeruginosa* cells are more resistant to the attraction and penetration of QAS reagents [44].

**Figure 5:**
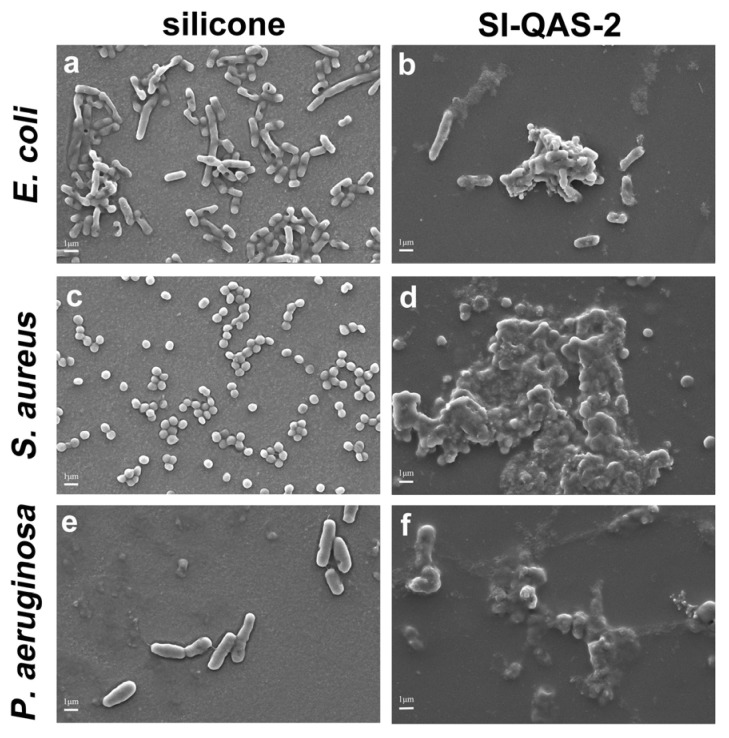
Morphologies of *E. coli*, *S. aureus*, and *P. aeruginosa* cells on the surfaces of silicone (**a**,**c**,**e**) and SI-QAS-2 (**b**,**d**,**f**).

A mechanism for the antibacterial activity of QAS has been reported and is described as follows. QAS adsorbs onto the surface of bacterial cells, with hydrophobic groups inserted into the lipid layer, thereby altering the permeability of the cell membrane and disrupting the membrane structure. This results in the subsequent leakage of intracellular substances as well as enzyme and protein denaturation, which affect cell metabolism and ultimately leads to the inactivation of bacterial cells [35,36]. Using an SEM, morphological changes in *E. coli*, *S. aureus*, and *P. aeruginosa* cells were observed after 30 min of incubation with SI-QAS-2 (Figure 5), and the antibacterial mechanism of this coating was inferred based on these observations. It can be seen from Figure 5 (a, c and e) that *E. coli* and *P. aeruginosa* cells exhibited their original rod shape while *S. aureus* cells maintained their inherent spherical shape, indicating that the three types of bacterial cell could grow normally on the silicone surface. In contrast, on the surface of SI-QAS-2 (Figure 5 (b, d and f)), all three bacterial cell types lost their original morphology and exhibited severe rupture and collapse. These results are consistent with the antibacterial mechanism of QAS in disrupting bacterial membranes.

### 3.4. Long-Term Bactericidal Activity of SI-QAS-2 Coating

**Figure 6:**
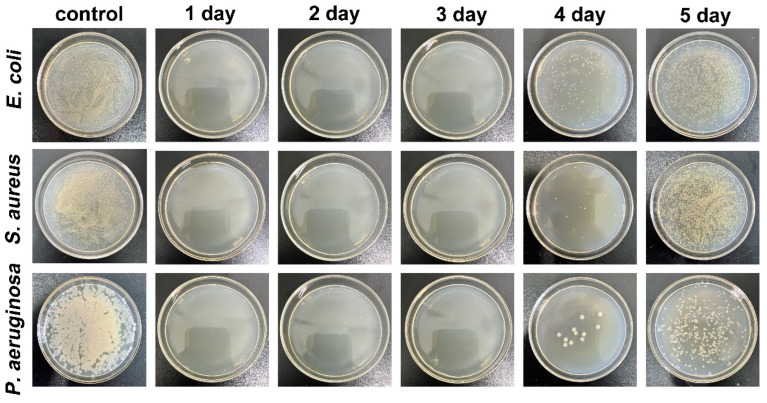
Images of bacterial colony on LB agar plate for *E. coli*, *S. aureus*, and *P. aeruginosa* after incubation with SI-QAS-2 for 1, 2, 3, 4, and 5 days.

To evaluate their effective bactericidal duration, SI-QAS-2 samples (1.0 cm × 1.0 cm) were placed into 24-well plates and exposed to a 10^6^ CFU/mL bacterial suspension for five days. In this test, the silicone samples were still used as the control group, and *E. coli*, *S. aureus*, and *P. aeruginosa* were used as typical pathogenic bacterial models. To maintain a constant bacterial concentration, the bacterial suspensions in each well were replaced daily. Figure 6 shows photos of LB agar plates obtained from different strains in different incubation periods. For all three bacterial types, SI-QAS-2 showed good antibacterial activity after four days, but after five days, there was a significant increase in viable bacterial colonies, indicating that the antibacterial effect had begun to decline.

**Figure 7:**
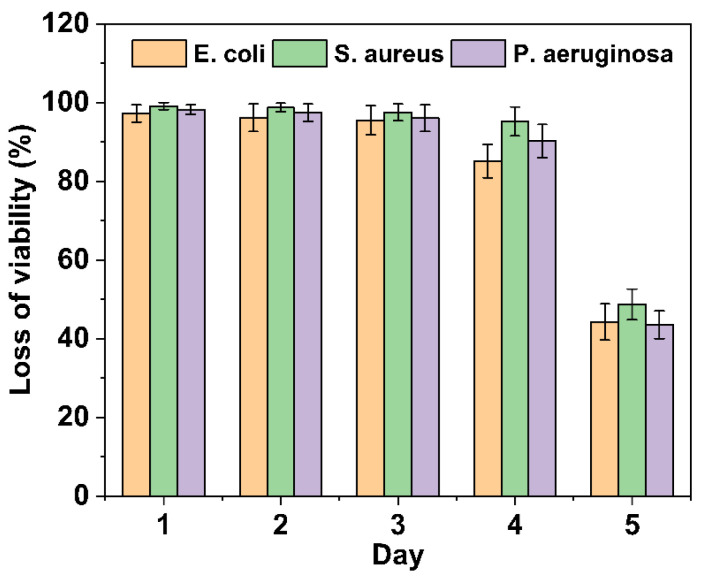
Long-term testing of loss of viability of *E. coli*, *S. aureus*, and *P. aeruginosa* incubated with SI-QAS-2 for 1, 2, 3, 4, and 5 days.

The quantitative results are shown in Figure 7. During the first three days of incubation with SI-QAS-2, the loss of viability of the three types of bacteria was higher than 95% in all cases, indicating that the coating could maintain high-level bactericidal ability over three days. After four days of incubation, the loss of *E. coli* and *P. aeruginosa* viability decreased slightly to 85.1% and 90.2%, respectively, while the loss of *S. aureus* viability was maintained at 95.2%. It was not until 5 days of incubation that there was a significant decrease in the loss of viability for the three types of bacteria, which dropped to 44.2%, 65.5%, and 43.5%, respectively. Long-term exposure to high concentrations of bacteria may result in the SI-QAS-2 surface being covered by dead cells and contaminated with intracellular substances, which may explain the reduction in the bactericidal ability of SI-QAS-2. Overall, the above indicates that SI-QAS-2 can maintain its bactericidal ability for a relatively long period (four days) in an environment of a high bacterial concentration (10^6^ CFU/mL). Normally, the bacterial concentration in urine or the urethra does not reach as high as 10^6^ CFU/mL [45], so the SI-QAS-2 coating can be expected to effectively prevent CAUTI after implantation.

## 4. Conclusions

In this study, a class of QAPU-vinyl polymers with two vinyl end groups and different QAS contents in their backbone was designed and successfully prepared. Using their vinyl end groups as active sites, the QAPU-vinyl polymers were covalently grafted onto SI-vinyl surfaces, resulting in a series of QAS-containing coatings. SI-QAS-2 was selected as the optimal coating based on consideration of stability, compatibility, and antibacterial properties. SI-QAS-2 had a surface QAS content of 2.1 × 10^16^ N^+^/cm^2^, which was much higher than the value required for effective bactericidal activity, 10^14^ N^+^/cm^2^, and the obtained SI-QAS-2 was demonstrated to have high bactericidal ability. After 30 min of contact with 10^6^ CFU/mL of *E. coli*, *S. aureus*, and *P. aeruginosa* cells, the survival ratios of these three bacteria decreased by 72.8%, 99.9%, and 98.9%, respectively. In addition, SI-QAS-2 could maintain a high bactericidal ratio of over 80% after being exposed to bacterial suspension containing 10^6^ CFU/mL of these three bacterial cells for 4 days. In summary, we developed an excellent contact-killing coating that demonstrates high affinity with silicone substrates, strong bactericidal ability, and the long-term maintenance of bactericidal activity. As a competitive antibacterial coating for silicone catheters, it has great potential for the prevention of CAUTI.

## Figures and Tables

**Table 1:** Molecular weight information for the PU-vinyl polymers with varied MDEA feeding.

PU-Vinyl Sample	Molar Ratio of Reactants in Feed				MDEA in PU ^a^ (mol%)	Molecular Weight ^b^		
	MDEA	BEPD	HDI	DC		Mn	Mw	PDI
1	0.1	0.9	1.1	0.11	12.2	6920	11,070	1.6
2	0.2	0.8	1.1	0.11	21.8	6500	9750	1.5
3	0.3	0.7	1.1	0.11	30.5	6150	9220	1.5
4	0.4	0.6	1.1	0.11	41.5	5980	8380	1.4

^a^ Percentage of MDEA units to total diol units in PU calculated from ^1^H NMR; ^b^ determined using GPC.

## Data Availability

The original contributions presented in this study are included in the article/Supplementary Material. Further inquiries can be directed to the corresponding author.

## Supplementary Materials

The following supporting information can be downloaded at https://www.mdpi.com/article/10.3390/polym17010017/s1, Figure S1: ^1^H NMR spectrum of N-bis(vinylbenzyl) diglycolamine; Figure S2: ^13^C NMR spectrum of N-bis (vinylbenzyl) diglycolamine; Figure S3: Mass spectrum of N-bis (vinylbenzyl) diglycolamine; Figure S4: (a) UV/Vis spectra of water solutions of sodium fluorescein with concentrations ranging from 0.1 μg/mL to 50 μg/mL and UV spectra of sodium fluorescein released from SI-QAS-1 (brown) and SI-QAS-2 (red) coating; (b) Standard curve of sodium fluorescein; Figure S5: FT-IR spectra of MDEA, HDI, BEPD, PU-vinyl-2, and QAPU-vinyl-2; Figure S6: XPS wide-scan spectra of SI-vinyl and SI-QAS-2 (a), C 1s spectrum of SI-vinyl (b), C 1s spectrum of SI-QAS-2 (c), and N 1s spectrum of SI-QAS-2 (d).
