# Supplementary material for: Covalent Grafting of Quaternary Ammonium Salt-Containing Polyurethane onto Silicone Substrates to Enhance Bacterial Contact-Killing Ability
Source: Polymers (Basel). 2024 Dec 25;17(1):17. doi: 10.3390/polym17010017 (PMC11723070; doi:10.3390/polym17010017)
Supplement: Supplementary file 1 [file polymers-17-00017-s001.zip › polymers-3362138-supplementary.pdf]

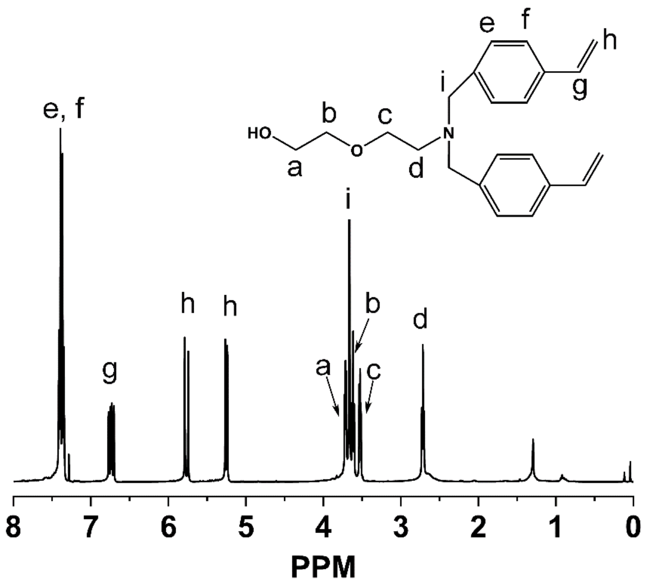

Figure S1. ^{1}H NMR spectrum of N-bis(vinylbenzyl) diglycolamine.

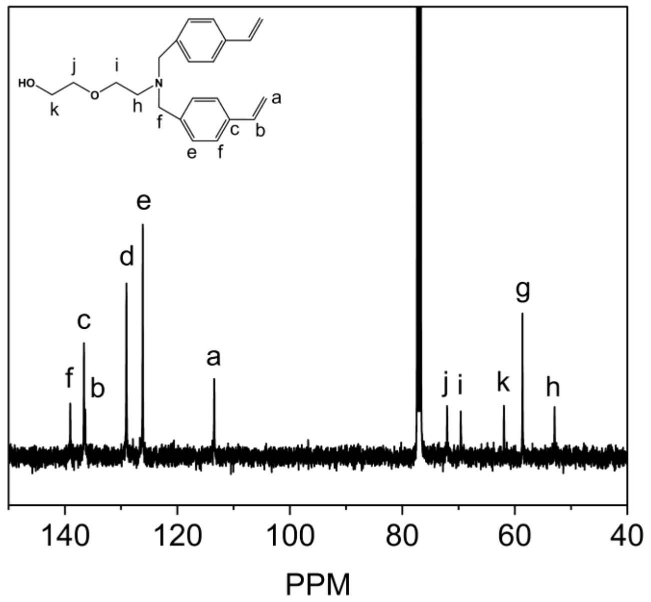

Figure S2. ^{13}C NMR spectrum of *N*-bis (vinylbenzyl) diglycolamine

Using $CDCl_3$ as solvent, the ^{13}C-NMR spectra of N-bis (vinylbenzyl) diglycolamine were measured as shown in **Figure S1**. 77.2 ppm is the solvent peak of $CDCl_3$. The peaks at 136.6, 126.1, 129.1 and 139.0 ppm are corresponding to four different carbon atoms of c, d, e, f in benzene ring, and the peaks at 113.4 and 136.4 ppm are contributed by the carbon atoms from vinyl group. The signal at 58.6 ppm (g) is assigned to the peak of benzyl carbon while the signal at 52.9 ppm (h) is attributed to the methylene carbon at α position of amino group. Other signals at 61.9, 69.6, and

72.0 ppm are assigned to the methylene carbon (k, i, and j) in the hydroxy arms.

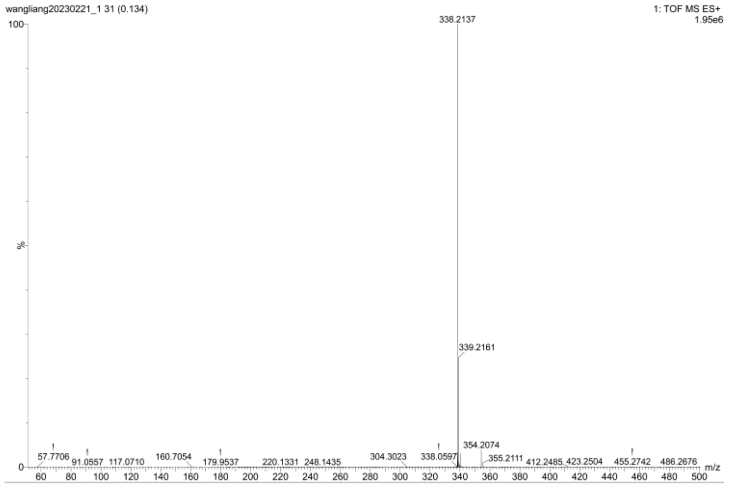

Figure S3. Mass spectrum of *N*-bis (vinylbenzyl) diglycolamine

HR-ESI-MS (positive mode) m/z: 338.2137 $[M+H]^+$ (calculated for $C_{19}H_{28}NO_2^+$, 338.2115).

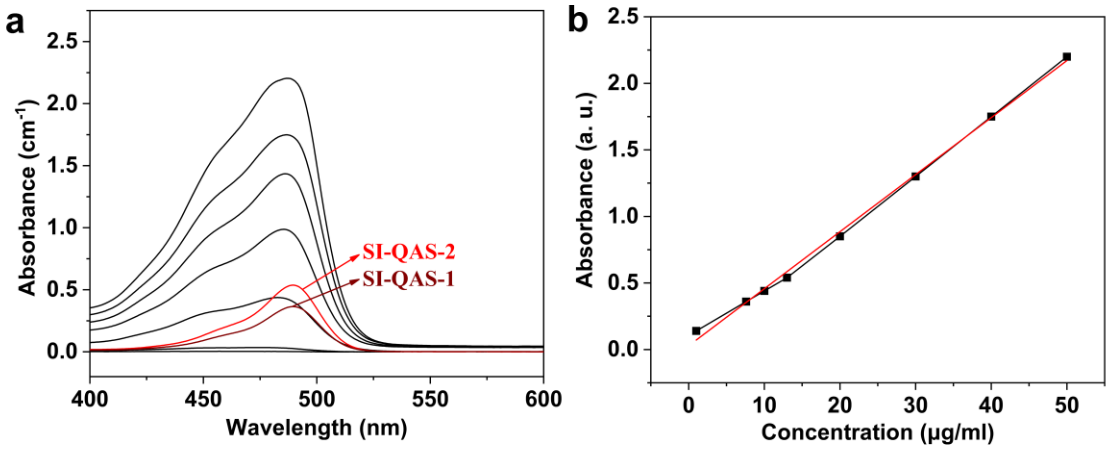

Figure S4. (a) UV/Vis spectra of water solutions of sodium fluorescein with concentrations ranging from 0.1 μg/mL to 50 μg/mL and the UV spectra of the sodium fluorescein released from the SI-QAS-1 (brown) and SI-QAS-2 (red) coating; (**b)** The standard curve of sodium fluorescein.

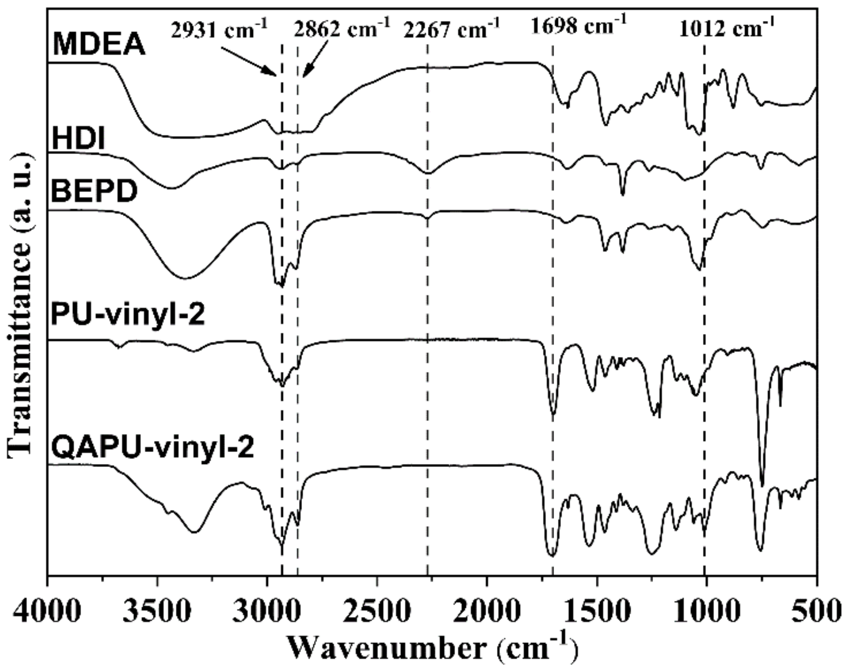

Figure S5. FT-IR spectra of MDEA, HDI, BEPD, PU-vinyl-2, and QAPU-vinyl-2.

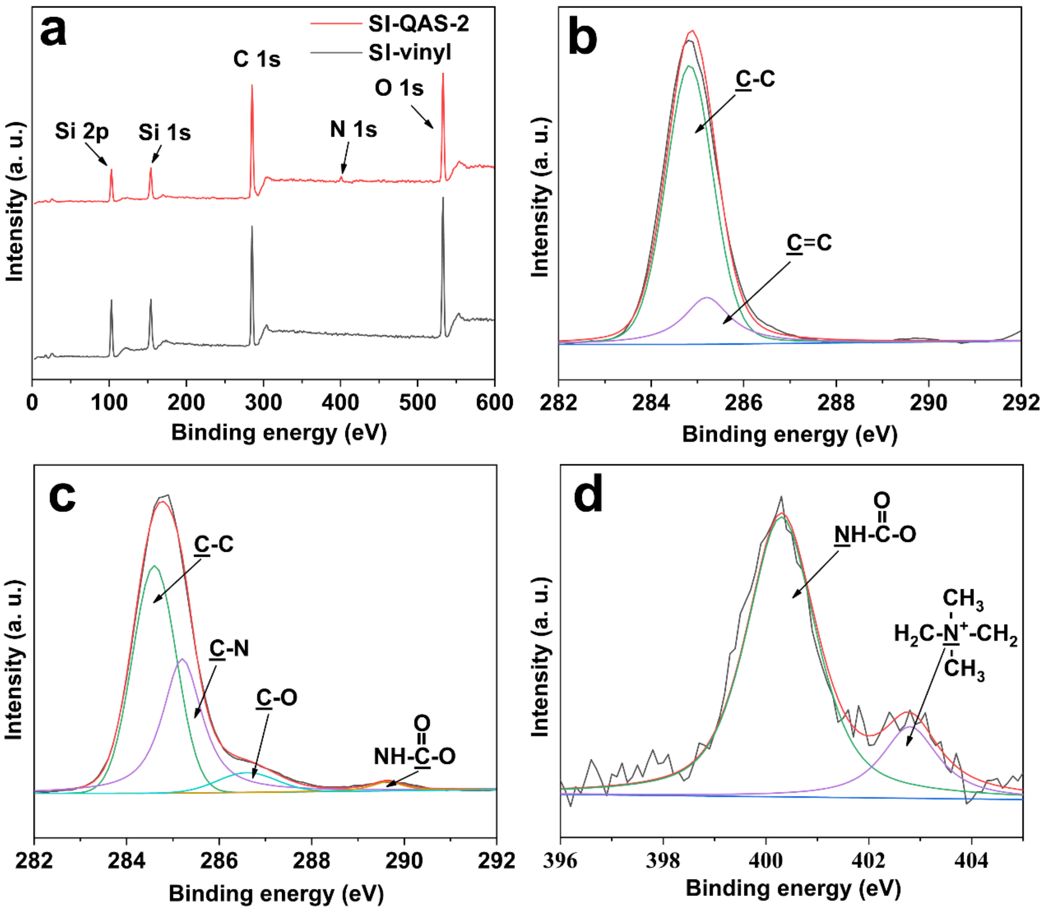

Figure S6. XPS wide scan spectra of SI-vinyl and SI-QAS-2 (a), C 1s spectrum of SI-vinyl (b), C 1s spectrum of SI-QAS-2 (c), and N 1s spectrum of SI-QAS-2 (d)
